# Radioimmunotherapy study of ^131^I-labeled Atezolizumab in preclinical models of colorectal cancer

**DOI:** 10.1186/s13550-022-00939-2

**Published:** 2022-10-28

**Authors:** Linhan Zhang, Sheng Zhao, Huijie Jiang, Rongjun Zhang, Mingyu Zhang, Wenbin Pan, Zhongqi Sun, Dandan Wang, Jinping Li

**Affiliations:** 1 Department of Nuclear Medicine, The First Affiliated Hospital of Harbin Medical University, Harbin, China; 2 Department of Radiology, The Second Affiliated Hospital of Harbin Medical University, Harbin, China; 3 Jiangsu Institute of Nuclear Medicine, Wuxi, China; 4 Department of Nuclear Medicine, Beijing Friendship Hospital, Affiliated to Capital Medical University, Beijing, China

**Keywords:** Radioimmunotherapy, Programmed cell death 1 ligand 1, Colorectal cancer, Cerenkov luminescence imaging, Molecular imaging

## Abstract

**Background:**

Programmed cell death 1 ligand 1(PD-L1) is overexpressed in many tumors. The radionuclide-labeled anti-PD-L1 monoclonal antibody can be used for imaging and therapy of PD-L1 overexpressing cancer. Here, we described ^131^I-labeled Atezolizumab (^131^I-Atezolizumab, targeting PD-L1) as a therapeutic agent for colorectal cancer with PD-L1 overexpression.

**Methods:**

^131^I-Atezolizumab was prepared by the Iodogen method. The expression levels of PD-L1 in different human colorectal cells were determined by flow cytometry, western blot and cell binding assay. The immunoreactivity of ^131^I-Atezolizumab to PD-L1 high-expressing cells was determined by immunoreactive fraction. The killing abilities of different concentrations of ^131^I-Atezolizumab on cells with high and low expression of PD-L1 were detected by the 3-(4,5-dimethylthiazol-2-yl)-2,5-diphenyltetrazolium bromide (MTT) method. Cerenkov luminescence imaging (CLI) and radioimmunotherapy (RIT) of ^131^I-Atezolizumab were performed on two human colorectal cancer models. The distribution and tumor targeting of ^131^I-Atezolizumab were evaluated by imaging. Tumor volume and survival time were used as indicators to evaluate the anti-tumor effect of ^131^I-Atezolizumab.

**Results:**

The expression level of PD-L1 in vitro determined by the cell binding assay was related to the data of flow cytometry and western blot. ^131^I-Atezolizumab can specifically bind to PD-L1 high-expressing cells in vitro to reflect the expression level of PD-L1. Immunoreactive fraction of PD-L1 high-expressing RKO cells with ^131^I-Atezolizumab was 52.2%. The killing ability of ^131^I-Atezolizumab on PD-L1 high-expressing cells was higher than that of low-expressing cells. CLI proved that the specific uptake level of tumors depends on the expression level of PD-L1. Effect of ^131^I-Atezolizumab RIT showed an activity-dependent tumor suppressor effect on RKO tumor-bearing mice with high PD-L1 expression. ^131^I-Atezolizumab (37 MBq) can improve the median survival time of mice (34 days), compared to untreated mice (27 days) (*P* = 0.027). Although a single activity(37 MBq) of ^131^I-Atezolizumab also inhibited the tumors of HCT8 tumor-bearing mice with low PD-L1 expression (*P* < 0.05), it could not prolong the survival of mice(*P* = 0.29).

**Conclusion:**

^131^I-Atezolizumab can be used as a CLI agent for screening PD-L1 expression levels. It may be used as a radioimmunotherapy drug target for PD- L1 overexpressing tumors.

**Supplementary Information:**

The online version contains supplementary material available at 10.1186/s13550-022-00939-2.

## Background

According to the latest data released by the International Agency for Research on Cancer, the colorectal cancer is the second leading cause of cancer deaths, accounting for 10% of the total number of cancers diagnosed and cancer-related deaths each year [1]. At present, more and more methods for the treatment of colorectal cancer have been developed, including surgery, radiotherapy and chemotherapy, molecular targeted therapy and immunotherapy, etc. However, the prognosis of end-stage colorectal cancer is still poor, and researchers are still looking for new treatment methods.

Programmed cell death 1 ligand 1(PD-L1) is highly expressed in many tumors, and monoclonal antibodies (mAb) to the programmed cell death 1/programmed cell death 1 ligand 1(PD-1/PD-L1) pathway have achieved good results in the treatment of malignant melanoma, non-small cell lung cancer, urothelial cancer and head and neck cancer [2], so PD-L1 is a good therapeutic target. However, it is only effective for colorectal cancers with microsatellite instability (MSI) and difficult to treat with conventional chemotherapy [3]. It had been suggested that external beam radiotherapy could up-regulate PD-L1 expression through the phosphoinositide 3-kinase/AKT and signal transducer and transcriptional activator 3 pathways [4]. External beam radiotherapy and combined with anti-PD-1-mAb or anti-PD-L1-mAb can produce an effective CD8^+^ T cell response, which can improve local tumor control, prolong survival and protect against tumor re-attack [4, 5]. However, radiotherapy is only conducive to the control of the primary site of the tumor and does not have a good effect on the metastatic disease of colorectal cancer that is easy to metastasize. Same as for external beam radiotherapy, the mechanism of action of radioimmunotherapy (RIT) is mainly driven by the delivery of ionizing radiation to tumor cells, therefore one may expect a similar effect on the expression of PD-L1. In a fluorescence imaging study of the patient-derived tumor xenograft (PDX) model in vivo, Wen found that the ^131^I-Cy5.5-anti-PD-L1-mAb probe showed prominent fluorescence intensity at the tumor site compared with the Cy5.5-anti-PD-L1-mAb probe. Correspondingly, histological analyses showed that PDXs of the ^131^I-Cy5.5-anti-PD-L1 group presented higher PD-L1 level than the Cy5.5-anti-PD-L1 group [6]. Ren used ^177^Lu-labeled anti-PD-L1-mAb for RIT. Analysis of changes in the immune microenvironment after treatment using flow cytometry showed an increase in CD45^−^/PD-L1 cells in tumors in the 3.7 MBq ^177^Lu-PD-L1-mAb group compared to the control group [7]. Pang found that ^131^I‐PD‐L1-mAb therapy was more effective than anti‐PD‐L1-mAb therapy in an immunocompetent murine model of triple-negative breast cancer [8]. Therefore, we attempted to treat human-derived colorectal cancer in tumor-bearing mice by RIT employing therapeutic radionuclides coupled with Atezolizumab.

Atezolizumab, a humanized mAb developed by Roche as a PD-L1 blocker, was approved for marketing by the US Food and Drug Administration (FDA) in 2016. It binds to PD-L1 on tumor cells and blocks its interaction with PD-1 on T cells and antigen-presenting cells, thereby relieving PD-1-mediated immunosuppression and promoting T cell attack on tumor cells. It has a promising future in the treatment of colorectal cancer [9]. In 2018, ^89^Zr-Atezolizumab PET imaging had been successfully used in human studies to assess PD-L1 status [10]. In a previous study, we used ^131^I-Atezolizumab as a Cerenkov luminescence imaging (CLI) agent to accurately assess PD-L1 expression in several types of colorectal cancer [11]. Therefore, we selected Atezolizumab as a targeting agent for RIT.

RIT is a potential treatment, which uses antigen and antibody interactions to bring radioactive therapeutic nuclides to the tumor site for intra-radiation therapy. The most commonly used radionuclide for RIT is β emitters (^131^I, ^177^Lu, ^90^Y), which have a long range in tissues to damage cells near the target. ^90^Y can be used to treat large masses, while ^177^Lu and ^131^I can be used to treat small residual lesions [12]. However, antibodies labeled with radioactive metals (such as ^177^Lu) will accumulate for a longer time in normal clearing organs (liver and kidney) and may cause toxicity [13, 14], so we focus on halogen-labeled antibodies. Considering the low cost and easy availability of the radionuclide ^131^I, we used ^131^I-labeled Atezolizumab (^131^I-Atezolizumab) to conduct RIT research. In our research, we prepared ^131^I-Atezolizumab for cell uptake, immunoreactive fraction and cytotoxicity studies in vitro, and we also conducted pharmacokinetic studies, CLI and RIT studies in vivo. This article is a preliminary study. Our study aims to investigate whether ^131^I-Atezolizumab could be used as RIT drug to target PD-L1 overexpressing colorectal cancer.

## Methods

### Cell culture

Human-derived colorectal cancer cell lines RKO, HCT8, DLD-1 and Caco-2 cells were obtained from the Cell Bank of the Chinese Academy of Sciences. Cells were cultured with Mem (Thermo Fisher Scientific, USA) or RPMI 1640 mediums (Biological Indus-tries, Israel) supplemented with 10% fetal bovine serum (Biological Indus-tries, Israel) and 1% penicillin/streptomycin (Beyotime Biotechnology, China) at 37 °C in 5% CO_2_. The medium was changed every two to three days. Cells in logarithmic growth phase were used for biological evaluation in vitro.

### PD‑L1 expression detection by flow cytometry

Flow cytometry was used to detect PD-L1 expression in cancer cells using a PE-labeled mouse anti-human PD-L1 antibody (Cat. #557924, BD Biosciences, USA). Briefly, RKO, DLD-1, Caco-2 and HCT8 cells were trypsin digested, and 1 × 10^6^ cells (100 μl) were incubated with 20 μl antibodies in the dark for 30 min at 4 °C. Meanwhile, the isotype control and background signal were provided by cells incubated with mouse IgG1 and phosphate-buffered saline (PBS), respectively. The fluorescence uptake of cancer cells was immediately assessed using a FACS Calibur flow cytometer (BD Biosciences, USA). FlowJo was used to perform a quantitative analysis of mean fluorescence intensity.

### PD‑L1 expression detection by western blot

Colorectal cancer cells of RKO, DLD-1, Caco-2, HCT8 at the logarithmic growth phase were lysed on ice for 30 min with RIPA (Beyotime Biotechnology, China). The cell lysates were centrifuged, and the protein concentration was detected by Coomassie Blue staining. Approximately 50 μg of cell lysates were electrophoresed on 8% SDS-PAGE and transferred to PVDF membranes. The membranes were incubated with antibodies of Rabbit anti-human PD-L1 (1:1000, ab205921, Abcam, UK) and GAPDH (1:1000, AF1186, Beyotime Biotechnology, China) at 4 °C overnight. After incubation with mouse anti-rabbit IgG-HRP antibody (1:1000, sc-2357, Santa Cruz Biotechnology, USA), the membranes were visualized using western blotting ECL reagent solution (Shanghai Share Biotechnology Co., Ltd., China).

### Radiolabeling

Atezolizumab (MPDL3280A) was purchased from MedChemExpress Company. Na^131^I was purchased from Chengdu Gaotong Isotope Co., Ltd. (China). Antibodies were labeled by the Iodogen method following a previously reported method [15]. Briefly, 100 μg Atezolizumab and 100 μl phosphate buffer (PB, 0.25 mol/L, pH 7.4) were added to a coating tube containing 50 μg Iodogen and mixed, and then, about 185-330 MBq Na^131^I was added and mixed. Let them react at room temperature for 15 min. 100 μl of PB (0.25 mol/L, pH 7.4) was added, and let them stand for 10 min, then stop the reaction. ^131^I-Atezolizumab was purified with PD-10 column (GE Healthcare, USA) using 20 mmol/L PBS containing 0.2% bovine serum albumin (BSA) as eluent. The radiochemical purity of the ^131^I-Atezolizumab was 96.18 ± 0.61% (*n* = 8) by trichloroacetic acid precipitation method.

### In vitro assays

#### Cell binding assay

^131^I-Atezolizumab cell binding assay was performed using RKO, DLD-1, HCT8 and Caco-2 cells. Each of cell suspensions (5 × 10^5^cells/tube, 100 μl, triplicate), PBS binding buffer containing 0.2% BSA (100 μl) and ^131^I-Atezolizumab (100 μl, 3.7 kBq, 2.79 ng) were mixed and incubated at 37 °C for 1 h. To measure nonspecific binding, a 200-fold molar ratio of non-radioactive Atezolizumab (100 μl, 558 ng) was used instead of the buffer. After the incubation, 700 μl of the binding buffer that had been cooled to 4 °C was added to each tube, and the supernatant was discarded by centrifugation. Then, each sample was counted in a gamma counter (Wizard 2480, PerkinElmer) and cell bound radioactivity (%) was calculated by (cell bound radioactivity – nonspecific binding radioactivity)/total radioactivity × 100.

#### Immunoreactive fraction

In order to evaluate the immunoreactivity of ^131^I-Atezolizumab, the immunoreactive fraction study was conducted with RKO cells. According to Lindmo et al. [16], different concentrations of cell suspension (100 μl), PBS binding buffer containing 0.2% BSA(100 μl) and ^131^I-Atezolizumab (100 μl, 3.7 kBq, 2.79 ng) were incubated at 37 °C for 1 h. To measure nonspecific binding, the above binding buffer solution was replaced by a non-radioactive Atezolizumab (100 μl, 558 ng) with a molar ratio of 200 times. The activity of the precipitated cells after centrifugation was measured with a gamma counter (Wizard 2480, PerkinElmer). The inverse of the specific cell bound activity was plotted against the inverse of the cell concentration. The data were fitted to a linear fit using least-squares linear regression. The inverse of immunoreactive fraction was represented by the regression line's Y-intercept.

#### Cell cytotoxicity assay

Cell cytotoxicity was assessed using a 3-(4,5-dimethylthiazol-2-yl)-2,5-diphenyltetrazolium bromide (MTT) assay. RKO and HCT8 cells were incubated in 96-well plates (8000 cells/well). After overnight incubation, the cells were adherent, and the supernatant was taken out. The cells in the treatment group were incubated in the medium containing different concentrations of ^131^I-Atezolizumab (29.6, 14.8, 7.4, 3.7, 1.85 MBq/ml,100 μl), and the cells in the control group were incubated in the medium containing non-radioactive Atezolizumab (2.2 μg/well) for 4 h. After the medium containing ^131^I-Atezolizumab and unlabeled Atezolizumab was aspirated, the complete medium was added. Then, the cells were incubated for another 3 days. MTT reagent (20 μl, 5 mg/ml) was added and incubated at 37 °C for 4 h. The formazan produced by living cells was dissolved in dimethyl sulfoxide (DMSO,150 μl/well), and the absorbance was measured at 490 nm using a microplate reader.

### Animal studies

5-6-week-old female Balb/c nu mice were obtained from Changzhou Cavens Laboratory Animal Co., Ltd. (Changzhou, China), and housed in an SPF-grade animal laboratory. The colon cancer RKO and HCT8 tumor models were established by injecting (3–5) × 10^6^ tumor cells into the armpits of mice. The thyroid tissue was sealed by drinking 0.5% sodium iodide solution one day before and during the imaging and treatment. All procedures were approved by the Institutional Animal Care and Use Committees of Jiangsu Institute of Nuclear Medicine.

### In vivo studies

#### Pharmacokinetic studies of ^131^I-Atezolizumab

Four normal Balb/c nu mice were injected with ^131^I-Atezolizumab (0.74 MBq,448 ng) into the tail vein, and tail vein blood samples were collected by tail-snip method at various time points (0.083, 0.25, 0.5, 1, 2, 4, 8, 12, 36, 48, 72, 168 h). Then, the radioactivity of blood samples was measured with a gamma counter (WIZARD 2480, PerkinElmer) and expressed as a percentage of injected activity per gram (%IA/g). Pharmacokinetic parameters of ^131^I-Atezolizumab in blood were calculated using DAS software version 2.1.1. Its pharmacokinetic parameters including terminal half-life (T_1/2z_), maximum concentration (C_max_), clearance rate (Cl) and area under the curve (AUC) were analyzed using a two-compartment model.

#### Small animal CLI

To evaluate the tumor distribution of ^131^I-Atezolizumab in models with different PD-L1 expression levels, ^131^I-Atezolizumab (37 MBq, 24.4 μg) was injected via tail vein. RKO and HCT8 tumor-bearing mice were subjected to CLI using the IVIS spectral imaging system (PerkinElmer, MA, USA) at different time points (1–9 days). Mice were placed in the supine position with continuous anesthesia. Tumor mean fluorescence intensity was measured on scanned images using Living Image® 4.5 software.

#### PD‑L1 expression detection by immunohistochemistry

RKO and HCT8 tumor tissues were embedded in paraffin blocks after fixation with tissue fixative. After routine deparaffinization and hydration of tissue sections, antigen retrieval was performed using 10 mmol/L citrate buffer, endogenous peroxidase was blocked with 3%H_2_O_2_ and nonspecific antigen blocking was performed with 3% BSA. The first antibody (1:200 ab205921, Abcam, UK) was added and incubated at 4 °C overnight. The second antibodies were incubated at room temperature for 1 h. DAB color development, hematoxylin staining, dehydrated and transparent were performed, and then, the specimen was mounted with neutral balsam. Brown-yellow staining was defined as positive. No coloration means negative, 0 points; light yellow means weak positivity, 1 point; brown-yellow means moderate positivity, 2 points; and tan means strong positivity, 3 points. The examinations results were performed in a double-blind manner by two independent investigators.

#### RIT

Thirty-five mice were used. The RKO model mice were randomly divided into 3 groups of 7 each, namely the RKO-37 MBq group, the RKO-11.1 MBq group and the RKO untreated group; HCT8 model mice were randomly divided into 2 groups of 7 each, namely HCT8-37 MBq group and HCT8 untreated group. In RKO-37 MBq group and HCT8-37 MBq group, ^131^I-Atezolizumab (37 MBq, 24.4 μg) was injected via tail vein. In the RKO-11.1 MBq group, a mixture of ^131^I-Atezolizumab (11.1 MBq, 7.32 μg) and non-radioactive Atezolizumab (17.08 μg) was injected via tail vein. Treatments were started when the tumor volume was about 50-100mm^3^. Tumor measurements and mice survival were recorded by a technician blinded to the grouping. Tumor growth was recorded by measuring tumors with vernier caliper twice a week and calculated using the following formula: tumor volume = (W)^2^ × (Y) × 0.52, where Y and W are the larger and smaller vertical diameters, respectively. Percentage increase in lifespan was expressed as (the median survival time of treated/the median survival time of control mice-1) × 100% [17]. The methods involved in the experiments were performed in accordance with relevant guidelines and regulations for animal experiments. The trial was terminated as long as at least one of the following criteria was met: tumor volume greater than 1500mm^3^; total body weight decreased by > 20%; severe ulceration or bleeding in the xenografted tumor; and abnormal behavior indicating pain or restlessness.

### Statistics

All experimental data conformed to normal distribution and were expressed as mean ± standard deviation. Statistical analysis was performed using SPSS 11 software (SPSS Inc., USA), and the comparison between the two groups was tested by Student’s t test. One-way ANOVA was used for comparison among multiple groups, and LSD t test was used for pairwise comparison. Survival data were estimated using the Kaplan–Meier method and compared using the log-rank test. *P* < 0.05 was considered significant.

## Results

### Expression levels of PD-L1 in human colorectal cancer cells

**Fig. 1:**
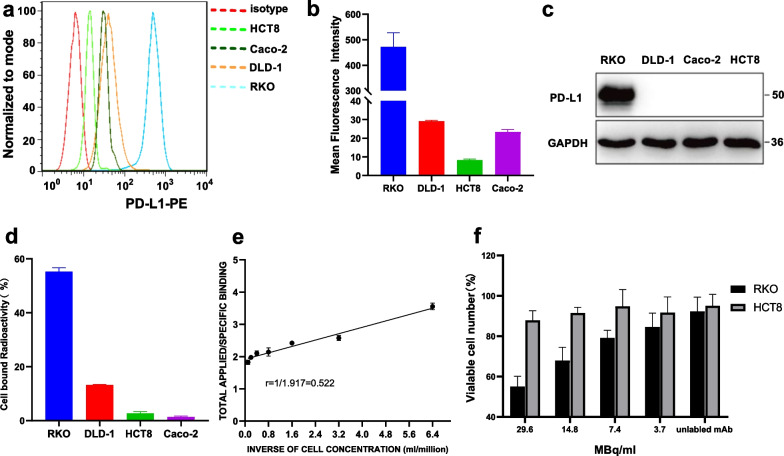
Analysis of PD-L1 expression and cytotoxicity of ^131^I-Atezolizumab on colorectal cancer cells. **a** Flow cytometry analysis of PD-L1 expression in different colorectal cancer cells (RKO, DLD-1, HCT8 and Caco-2). **b** FlowJo was used to conduct a quantitative analysis of mean fluorescence intensity. **c** PD-L1 (50 kDa) expression in cancer cells was analyzed using a western blot with GAPDH (36 kDa) serving as the internal control. **d** Cell binding assay of ^131^I-Atezolizumab. **e** The immunoreactive fraction of ^131^I-Atezolizumab and RKO cells. **f** Cytotoxicity of ^131^I-Atezolizumab

1. The relative expression levels of PD-L1 in four colorectal cancer cell lines (RKO, DLD-1, HCT8 and Caco-2) were determined by flow cytometry (Fig. 1a, b). The RKO cell line expressed higher levels of PD-L1, and the other three cell lines showed lower levels of PD-L1 expression. The mean fluorescence intensity of the four cells was statistically different (*P* < 0.001), and the mean fluorescence intensity of RKO cell and the other three cells was statistically different (*P* < 0.001), while there was no statistical difference between DLD-1 and HCT8, DLD-1 and Caco-2, HCT8 and Caco-2 (*P* = 0.380, 0.803, 0.521). The average fluorescence intensity of RKO cells was over 14 times higher than that of the other three cell lines.
2. The PD-L1 expression level in four different cancer cells (RKO, DLD-1, Caco-2 and HCT8) was also tested by western blot (Fig. 1c, Additional file 1: Fig. S1c). RKO cells highly expressed PD-L1, while the other three cells hardly expressed PD-L1. Since the expression levels of PD-L1 in Caco-2, HCT8 and DLD-1 cells were much lower than that in RKO cells, their expression levels could not be detected by western blot.
3. The cell binding rates of RKO, DLD-1, HCT8, Caco-2 cells to ^131^I-Atezolizumab were (55.28 ± 1.41) %, (13.25 ± 0.19) %, (2.13 ± 1.30) %, (1.39 ± 0.34) %, and the difference was statistically significant (*P* < 0.001). There was no significant difference between HCT8 cells and Caco-2 cells (*P* = 0.382), but there was significant difference between the other two groups (*P* < 0.001) (Fig. 1d). Immunoreactive fraction of RKO cell with ^131^I-Atezolizumab was 52.2% (Fig. 1e).

### Cytotoxicity of ^131^I-Atezolizumab in RKO and HCT8 Cell

The viability of RKO cells and HCT8 cells after treatment with different radioactive activities (29.6, 14.8, 7.4, 3.7 MBq/ml) of ^131^I-Atezolizumab was shown (Fig. 1f). ^131^I-Atezolizumab had little effect on HCT8 cells. Non-radioactive Atezolizumab also had no obvious killing effect on RKO cells and HCT8 cells. The killing effect of ^131^I-Atezolizumab on RKO cells increased in an activity-dependent manner. At an activity of 29.6 MBq/ml, the percentage of surviving cells decreased to (55.07 ± 5.09) %. The killing ability of ^131^I-Atezolizumab on RKO cells and HCT8 cells was significantly different (*P* < 0.001).

### ^131^I-Atezolizumab pharmacokinetics

The pharmacokinetic parameters of ^131^I-Atezolizumab in healthy nude mice are shown in Table 1.

**Table 1:** Pharmacokinetic parameters of ^131^I-PD-L1-mAb

Parameters	
*C*_max_(%IA/g)	47.83 ± 22.38
Cl(ml/h)	0.22 ± 0.07
AUC_0→∞_(%IA/g*h)	733.09 ± 125.00
T_1/2Z_(h)	67.81 ± 21.54
MRT_0→∞_(h)	78.53 ± 21.79

*C*_max_ maximum concentration, Cl clearance rate, AUC area under curve, T_1/2Z_ elimination half-life, MRT mean residence time

### CLI of ^131^I-Atezolizumab

**Fig. 2:**
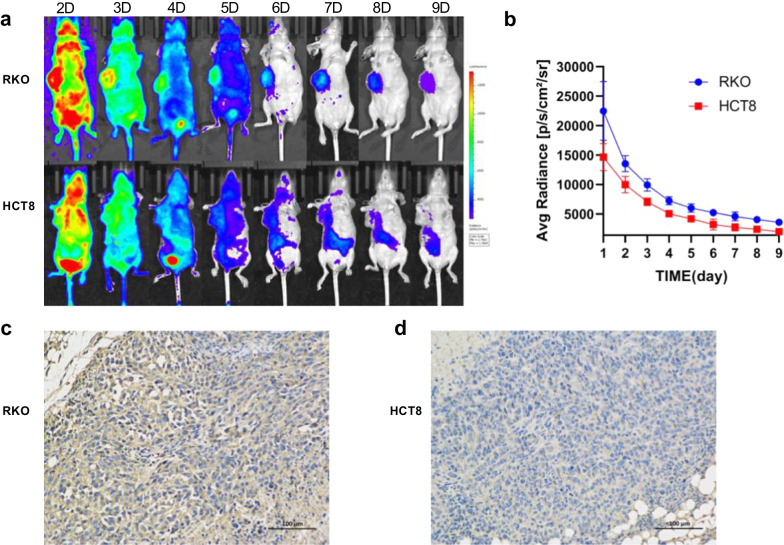
CLI and immunohistochemistry. **a** CLI of ^131^I-Atezolizumab in RKO model and HCT8 model at each time point. **b** Quantification of the average fluorescence intensity of CLI. **c**, **d** Immunohistochemical images of RKO model and HCT8 model

CLI technique was used to study the behavior of ^131^I-Atezolizumab in RKO and HCT8 tumor-bearing mice. Figure 2a shows representative images from 2–9 days of injection of ^131^I-Atezolizumab. After injection of ^131^I-Atezolizumab, the intratumoral fluorescence intensity gradually decreased with time. The mean fluorescence intensity of tumor in RKO tumor-bearing mice was 1.36–1.8 times higher than that in HCT8 tumor-bearing mice from 1 to 9 days after injection of ^131^I-Atezolizumab. There was a statistically significant difference in the mean fluorescence intensity between the two at each time point. (*P* = 0.029, 0.01, 0.003, 0.003, 0.007, 0.049, 0.046, 0.032, 0.022) (Fig. 2b).

### Immunohistochemistry

Figure 2c, d shows typical pictures of PD-L1 immunohistochemistry in RKO and HCT8 tissues. Both RKO and HCT8 tumor tissues express PD-L1, and the expression level of RKO tumor tissues was higher than that of HCT8 tumor tissues.

### Therapeutic efficacy

**Fig. 3:**
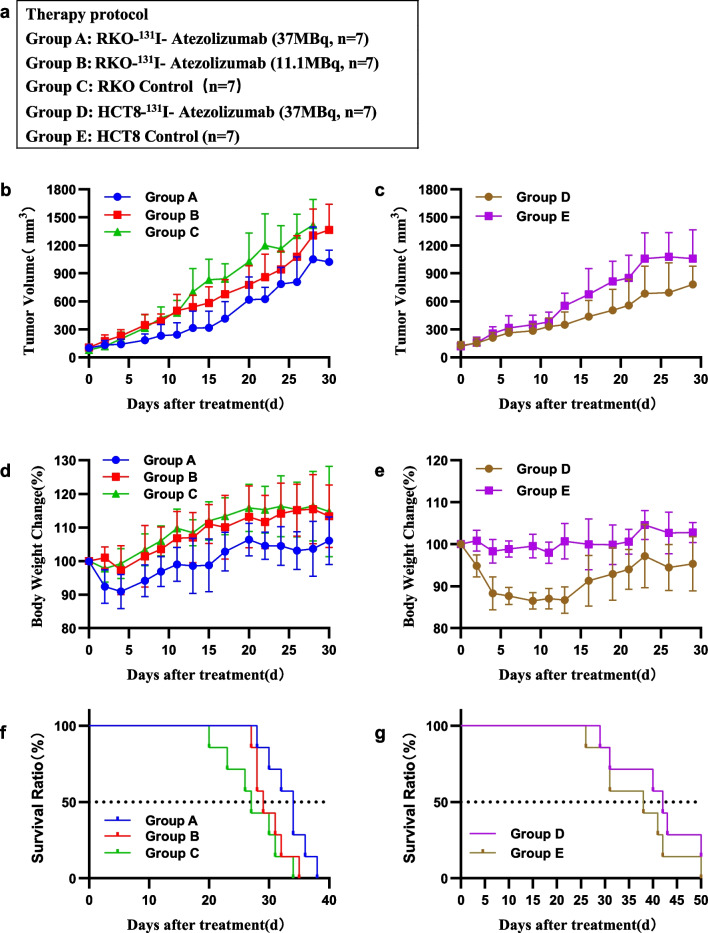
Effectiveness of ^131^I-Atezolizumab RIT on RKO model and HCT8 model. **a** Design of therapy protocol. **b**, **c** Tumor volume. **d**, **e** Body weight change. **f**, **g** Survival curve

The changes in tumor volume, weight and survival in different treatment modes of RKO and HCT8 subcutaneous tumor models are shown in Fig. 3 and Table 2.

**Table 2:** Mouse survival time

Model	Treatment modality	Survival		
		Median survival time(d)	*P* value	Lifespan (%)
RKO	^131^I-Atezolizumab (11.1 MBq)	29	0.326	7.4
	^131^I-Atezolizumab (37 MBq)	34	0.027	25.92
	Normal Control	27	–	–
HCT8	^131^I-Atezolizumab (37 MBq)	42	0.290	10.52
	Normal Control	38	–	–

*P* values were estimated between the experimental and normal control groups by log-rank test, *P* < 0.05 indicated significance

Percentage increase in lifespan was expressed as (the median survival time of treated/the median survival time of control mice-1) × 100%

Tumor volume increased with time in the untreated control group and the ^131^I-Atezolizumab treatment group. In the RKO model, the growth rate of tumor volume decreased with increasing activity. The mean tumor volume in the 11.1 MBq group at 15 days of treatment was (580.62 ± 187.50) mm^3^, which was statistically significant compared with the (865.35 ± 213.06) mm^3^ in the untreated group (*P* = 0.017); there was no significant improvement in the median survival of mice (*P* = 0.326). At 9 days of treatment, the mean tumor volume (231.37 ± 121.30) mm^3^ in the 37 MBq group was significantly different from the untreated group (422.51 ± 117.66) mm^3^ (*P* = 0.004). The median survival of mice was shortened by 7 days (*P* = 0.027). In the HCT8 model, the mean tumor volume (350.29 ± 136.73) mm^3^ in the 37 MBq group at 13 days of treatment was statistically different (*P* = 0.022) compared with the untreated group (553.39 ± 134.40) mm^3^, and the median survival of mice was not significantly improved (*P* = 0.290).

The body weight of all radionuclide-treated mice decreased with the activity administered and was less than 20% of the initial body weight. The animals did not experience abnormal behavior in pain or restlessness during the treatment.

## Discussion

Surgery is considered to be the first-line treatment for resectable colorectal cancer without metastasis. However, about 36% of patients were diagnosed with regional lymph node metastasis at the time of discovery and 22% of patients had distant metastases [18]. Clinically, a variety of methods are used to treat metastatic colorectal cancer, but the five-year survival rate is 14.7–72.2% [18, 19]. Therefore, it is necessary to develop appropriate new treatments. For patients who do not respond to conventional therapies, can RIT be used as an alternative therapy? Because a single activity can direct therapeutic radionuclides to primary tumors and metastatic lesions (including lymph nodes, lungs, liver, etc.). Depending on the range and type of radiation emitted by the radionuclide, RIT can be targeted for delivery to kill antigen-positive tumor cells. At the same time, the “cross-fire” effect can be exploited, which can kill antigen-negative tumor cells adjacent to cells expressing the target antigen. PD-L1 can be overexpressed in a variety of tumors and is an ideal therapeutic target. Traditional external beam radiation therapy is indicated for the treatment of local lesions, while RIT offers the possibility of treating localized, metastatic or diffuse tumors. Radiation killing mediated by anti-PD-L1-mAb may also have some advantages over radiation killing from external beam. In addition, radiation can increase the expression level of PD-L1 on the surface of tumor cells, promote the combination of ^131^I-PD-L1-mAb and cells and inhibit tumor growth. Therefore, it is very interesting to explore ^131^I-PD-L1-mAb RIT. We found that ^131^I-Atezolizumab RIT could inhibit the growth of PD-L1 high-expressed colorectal cancer tumors. We detected the expression level of PD-L1 and predicted the effect of RIT by ^131^I-Atezolizumab CIL.

PD-L1 expressed by tumor cells is a potential biomarker for predicting anti PD-1/PD-L1 immunotherapeutic responses [20]. At present, the main detection method to determine tumor PD-L1 expression is immunohistochemistry. On the one hand, this method has many disadvantages such as strong invasiveness, difficulty in sample collection and inconsistent positive criteria [21, 22]. On the other hand, PD-L1 expression levels are dynamic and heterogeneous [23], and biopsy at a single time point provides limited information throughout the treatment regimen. So we need a noninvasive but more accurate detection method. Many recent preclinical studies had demonstrated that real-time data on PD-L1 expression at the lesion site can be obtained through optical imaging [24, 25], positron emission computed tomography (PET) [26], single-photon emission computed tomography (SPECT) [27] and magnetic resonance imaging (MRI) [28]. Thus, these imaging methods have important advantages in detecting PD-1/PD-L1. Although compared to other tests, radionuclide CLI is often affected by tissue depth, organ size and thickness. However, it is inexpensive, simple and efficient to operate, and it takes only 5 min to obtain real-time data from 3 to 5 mice simultaneously. We found that the results of the radionuclide CLI were consistent with PD-L1 expression in tumor cytology and tumor tissue.

As far as we know, in mice model of human-derived colorectal cancer, this is the first article to study the effect of RIT with PD-L1 as the target. Most of the previous research objects were mouse tumor models [6–8, 29], and the RIT effect of ^131^I- or ^177^Lu-labeled PD-L1-mAb in murine tumors had been determined. However, the purpose of treatment ultimately returns to the study of human tumors. There were many previous studies on radioimmunization of human-derived tumors targeting PD-L1 [30–37]. However, these studies focused on PD-L1-targeted molecular imaging rather than RIT. PD-L1 is an immune checkpoint that is susceptible to the immune microenvironment. The physiological process is complex. So in this study we only tentatively explored whether RIT can be effective for subcutaneous human-derived colorectal cancer with high expression of PD-L1. The high expression of PD-L1 in tumor cells is an indicator of the more aggressive cancer phenotype of tumors [38, 39], and we can use this feature to conduct research. Through the observation of ^131^I-Atezolizumab CLI in RKO and HCT8 tumor-bearing mice, we found that the radionuclides in RKO tumors with high expression of PD-L1 had a long aggregation time. This makes RIT possible. By monitoring tumor size and survival, we found that RIT effect of the RKO model with high- expression of PD-L1 was better than that of the HCT8 model with low-expression of PD-L1, which was consistent with the study of MTT in vitro. Thus, ^131^I-Atezolizumab CLI has potential value in predicting the effects of RIT targeting PD-L1.

In our study, we found a significant difference in PD-L1 expression between RKO and HCT8 cells in vitro, but a smaller difference in RIT in vivo, and ^131^I- Atezolizumab (37 MBq) also inhibited HCT8 tumors with low PD-L1 expression. Our analysis suggested the following two causes. On the one hand, the long circulation time of ^131^I-labeled whole antibodies in vivo increased the residence time of the drug in the tumor; on the other hand, the effect of RIT was influenced not only by the level of cell surface antigen but also by tumor-specific binding, perfusion, vascular distribution, vascular permeability and plasma half-life [40]. Due to the different growth rates of tumors and the body conditions of mice in the two models, the treatment effects were difficult to compare. However, we still found differences between the two and the untreated group. The high activity of ^131^I-Atezolizumab had a significant inhibitory effect on RKO subcutaneous tumors (*P* = 0.004) and had a certain killing ability for HCT8 subcutaneous tumors (*P* = 0.022). In our study, because we utilized Atezolizumab that can cross-react with murine PD-L1, nonspecific isotype control was not introduced. Atezolizumab binds not only to tumors but also to other organs expressed PD-L1 such as the spleen, while nonspecific antibodies do not bind to PD-L1. This condition will result in inconsistent distribution of Atezolizumab and isotype antibodies in vivo, which may bias the results [41].

There are many deficiencies in this paper. Firstly, the labeled antibody is a full antibody, which has the disadvantages of longer cycle time, slower clearance and easy radiation damage to healthy tissues. Secondly, because we wanted to associate the results of ^131^I-Atezolizumab CLI with RIT, excess of cold antibody was not applied to animal studies, which represents a limitation. Thirdly, in the study of preparing ^131^I-Atezolizumab by Iodogen method, we found that its immune activity decreased when Atezolizumab was labeled with iodine (radio and non-radio) due to the particularity of Atezolizumab structure. If the activity of Na^131^I increased further, it will cause changes in the three-dimensional structure of Atezolizumab, resulting in ^131^I-Atezolizumab being prone to precipitation. Therefore, immunoreactive fraction of ^131^I-Atezolizumab and RKO cells was low. The low immunoreactive fraction is also a limitation, which may preclude from the approval for clinical use in some countries. Fourthly, due to the limitation of experimental funding, this study lacks classical biodistribution experiments. Finally, since our model could only be built using immunodeficient mice without thymus, it could not effectively induce CD8^+^T cell response, and the immune effect was limited. This study mainly explored the role of ^131^I-Atezolizumab in the treatment of PD-L1 overexpression of human colorectal cancer tumors. If a humanized model of high expression of PD-L1 with complete immune function can be established, further coordination with the immune effect of PD-L1 will play a better therapeutic role. This had been confirmed in research on murine tumors [6, 8]. If the study of ^131^I-Atezolizumab targeted internal irradiation therapy was carried out with Micro SPETCT/CT, the distribution of each organ would be more intuitively and accurately observed, which would be conducive to the research and clinical translation of internal radiation therapy. Although there are many deficiencies in our study, we still believe that the RIT effect of PD-L1 as a target in human-derived tumors is feasible, which will provide a basis for clinical application. If this method can be promoted, it will greatly save patients the cost of medical treatment because antibodies are expensive and RIT requires less antibodies. RIT is a targeted internal radiation therapy that inhibits the growth of PD-L1-overexpressing tumors through a single intravenous injection, which provides new insights into the treatment of human colorectal cancer.

## Conclusions

^131^I-Atezolizumab has inhibitory effect on PD-L1 high-expression tumors and may be a candidate drug for tumor diagnosis and treatment.

## Supplementary Information

**Additional file 1. Fig. S1c**. The raw data of Western blot experiment.

## Data Availability

All data generated or analyzed during this study are included in this published article.

## Abbreviations

PD-L1: Programmed cell death 1 ligand 1
PD-1: Programmed cell death 1
RIT: Radioimmunotherapy
CLI: Cerenkov luminescence imaging
mAb: Monoclonal antibodies
^131^I- PD-L1-mAb: ^131^I-labeled PD-L1 monoclonal antibodies
MTT: 3-(4,5-Dimethylthiazol-2-yl)-2,5-diphenyltetrazolium bromide
DMSO: Dimethyl sulfoxide
PET: Positron emission computed tomography
SPECT: Single-photon emission computed tomography
MRI: Magnetic resonance imaging
PB: Phosphate buffer
PBS: Phosphate-buffered saline
BSA: Bovine serum albumin
PDX: Patient-derived tumor xenograft
