# Supplementary material for: Radioimmunotherapy study of 131I-labeled Atezolizumab in preclinical models of colorectal cancer
Source: EJNMMI Res. 2022 Oct 28;12:70. doi: 10.1186/s13550-022-00939-2 (PMC9616992; doi:10.1186/s13550-022-00939-2)

**Radioimmunotherapy study of ^131^I-labeled Atezolizumab in preclinical models of colorectal cancer**

Linhan Zhang^1，2^, Sheng Zhao^2^, Rongjun Zhang^3*^, Huijie Jiang^2*^, Mingyu Zhang^4*^, Wenbin Pan^2^, Zhongqi Sun^2^, Dandan Wang^2^, Jinping Li^2^

**Fig. S1c** The raw data of Western blot experiment. We checked the picture results of the western blot experiment, and did not edit the data arrangement, but only cropped the picture height and width to make the image more beautiful. For this reason, we provide the original image before cropping, as follows：


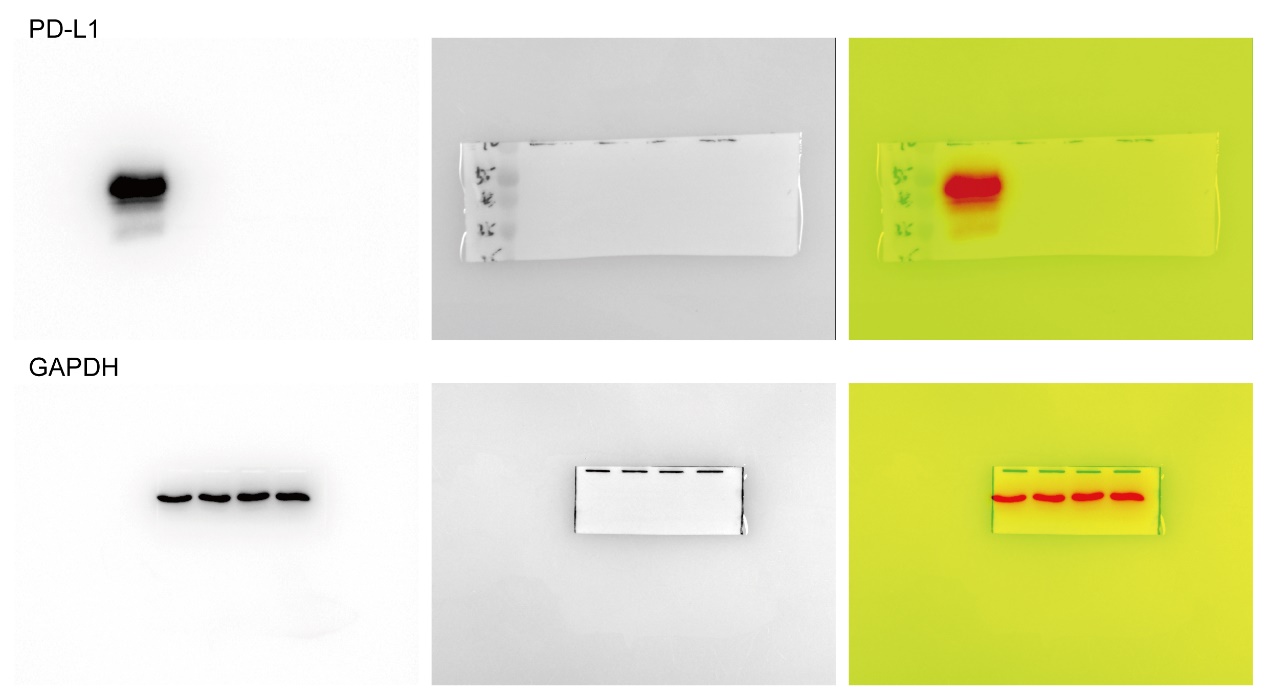

Supplement: Supplementary file 1 — Additional file 1. Fig. S1c. The raw data of Western blot experiment. [file 13550_2022_939_MOESM1_ESM.docx]
